# Current and potential contributions of community pharmacy teams to self-harm and suicide prevention: A qualitative interview study

**DOI:** 10.1371/journal.pone.0222132

**Published:** 2019-09-09

**Authors:** Hayley C. Gorton, Donna Littlewood, Christine Lotfallah, Matthew Spreadbury, Kai Ling Wong, Patricia Gooding, Darren M. Ashcroft

**Affiliations:** 1 Division of Pharmacy & Optometry, School of Health Sciences, Manchester Academic Health Science Centre, University of Manchester, Manchester, United Kingdom; 2 NIHR Greater Manchester Patient Safety Translational Research Centre, Manchester Academic Health Science Centre, University of Manchester, Manchester, United Kingdom; 3 School of Applied Sciences, University of Huddersfield, Huddersfield, United Kingdom; 4 Division of Psychology & Mental Health, School of Health Sciences, Manchester Academic Health Science Centre, University of Manchester, Manchester, United Kingdom; Institute of Mental Health, SINGAPORE

## Abstract

**Background:**

Suicide prevention is a global priority. Despite the focus on primary care in suicide prevention, little is known about the contributory role of community pharmacists and nothing about the role of the wider community pharmacy team in this area. We aimed to explore the current and potential role of community pharmacy teams in self-harm and suicide prevention.

**Methods:**

We conducted one-to-one semi-structured qualitative interviews with community pharmacy staff (pharmacists, pre-registration pharmacists, pharmacy technicians, dispensing/pharmacy assistants, delivery drivers) in the North West of England, UK. We identified themes from the interview transcripts through an iterative process of inductive thematic analysis.

**Results:**

We conducted twenty-five interviews with community pharmacy staff. Many described examples of helping those who were contemplating suicide or self-harm. No participants had received suicide prevention training. We identified six themes. The first two themes *(i) Relationship with Patient* and *(ii) Pharmacy environment* were seen as facilitators, which, if supported by *(iii) Training*, could underpin the final three themes: *(iv) Opportunities for contact*, *(v) Facilitated referral pathway and (v) Restricting access to means*. The distinct lack of training should be overcome with evidence-informed training. Referral pathways should be clear and enable direct and accessible referral by community pharmacy teams. There are opportunities for existing pharmacy services and schemes to be adapted to maximise suicide and self-harm prevention activities. Pharmacy teams did not identify themselves to have a clear role in restricting access to medication.

**Conclusions:**

Pharmacy teams already support patients in relation to self-harm and suicide, often relying on their personal experience in the absence of formal training. With the implementation of evidence-informed training and clear referral pathways, this could be done in a more effectively.

## Introduction

Over 800,000 people die worldwide by suicide every year [1] and in 2017 there were 5,821 registered suicides in the UK alone [2]. The World Health Organization (WHO) have pledged to reduce the rate of suicide by 10% by 2020 [1], and the UK Government have adopted the same target [3]. In recent years, there has been a series of UK Government reports and policies that aim to reduce suicide deaths [3–5]. Much of the focus relates to primary care, specifically targeting General Practitioners (GPs) and their support staff [4, 5]. The role of community pharmacy teams is not explicitly included in the aforementioned documents but is being mooted in some recommendations. For example, in the Public Health England “Local suicide prevention planning” resource, pharmacists are named as “partners in effective suicide prevention” [6]. In the Scottish Government’s 2018 suicide prevention plan, “Every Life Matters”, NHS pharmacists are specified as healthcare professionals who should undertake mandatory suicide prevention training, which will be available from May 2019 [7]. Pharmacy staff are also named as frontline clinical professionals in the National Collaborating Centre for Mental Health/Health Education England (NCCMH/HEE) Suicide and Self-harm Competency Frameworks, published in 2018 [8]. Despite these acknowledgements, the vision or expectation of the role of pharmacy teams has not been described.

With 1.6 million attendees every day in England alone, community pharmacies are the most visited healthcare provider [9]. Community pharmacies offer person-centred services that are delivered by the pharmacist, such as the Medicines Use Review (MUR) and the New Medicines Service (NMS). The MUR provides resource for pharmacists to undertake a structure medication review with the patient. The NMS enables pharmacists to follow-up patients on two additional occasions after an initial conversation at point of dispensing a new medication, to discuss compliance, adverse events and response to treatments. Currently, the service is limited to medicines prescribed for distinct set of conditions, none of which are mental health conditions [10]. The Quality Payments Scheme (QP) is a new way of remunerating community pharmacies that meet certain clinical effectiveness, patient safety and patient experience criteria [11]. One criterion is for pharmacies to become a level one Healthy Living Pharmacy (HLP), which requires them to undertakeadditional activities in the remit of public health. In 2018, the National Institute for Health and Care Excellence (NICE) issued guidance on community pharmacies promoting health and wellbeing [12], thus endorsing pharmacy as a public health resource tailored to the needs of the local community. A scoping review concluded that research on the remit and role of pharmacists and pharmacy teams in suicide prevention is scarce, and mainly limited to surveys [13]. A thematic analysis of responses to open-ended survey questions answered by pharmacists in Australia and Canada reported that pharmacists have a role in caring for people at risk of suicide, particularly by referral and triage, and directly supporting people with suicidal thoughts and behaviours [14]. Until now, there has been no published research on the role of community pharmacy teams in suicide awareness or prevention in the UK, and just one study globally that considers the role of the entire pharmacy team [15]. We therefore aimed to explore the current and potential role of community pharmacy teams in suicide prevention from the perspectives of members of those teams.

## Materials and methods

We conducted a qualitative study consisting of semi-structured, face-to-face interviews with 25 individuals who work within a community pharmacy based in Greater Manchester, Merseyside or West Yorkshire, UK.

### Ethics, consent and permissions

Ethical approval for this study was granted by the Health Research Authority (236561) and from The University of Manchester Research Ethics Committee (2017-2702-4542). All interviewers had completed training in qualitative research interviewing and attended a suicide prevention training course, in case any distress to the participants occurred.

### Recruitment and sampling

Participation was open to individuals currently employed in a community pharmacy, aged 18 years or over, and who spoke fluent English. We employed a purposive sampling strategy, which sought to ascertain views from those with different job roles in the community pharmacy team. This included pharmacy professionals who are registered with the regulator, the General Pharmaceutical Council (GPhC) i.e. pharmacists and pharmacy technicians. Pre-registration trainees (individuals in training year prior to registration but following the completion of a Master of Pharmacy degree) were included as were other staff who are typically trained but not registered. This included dispensing or healthcare assistants, and home delivery drivers. We included those who worked in different pharmacy settings such as those located on shopping streets, in a shopping centre or co-located with a GP surgery; and a mixture of those working for independent pharmacies and pharmacy chains. Participants were recruited to the study via email invitations that were distributed by professional pharmacy networks, and poster advertisements were circulated on social media websites such as Twitter.

### Procedure

All participants provided written consent prior to the interview, and were reimbursed for their time and travel expenses. Interviews lasted between 8 minutes and 64 minutes, and were conducted in a private setting either at the University of Manchester or at the participants’ workplace. Interviews were audio-recorded and transcribed verbatim, at which point identifiable data was omitted. On conclusion of the interview, participants were asked to complete a questionnaire containing demographical information which was used to characterise the sample.

### Interviews

Semi-structured, face-to-face interviews were conducted on a one-to-one basis by four members of the research team (HCG, CL, MS, KLW). A topic guide (S1 File) was used to inform the interviews, which covered four key areas: 1) general awareness of suicide and self-harm, and local sources of support, 2) previous experience of supporting a patient who had thoughts of suicide or self-harm or who had self-harmed, 3) views on the roles of community pharmacy teams in suicide and self-harm awareness and prevention, 4) views on training needs of community pharmacy teams on suicide and self-harm. Use of a topic guide helped to ensure that interviews generated data relating to the same key areas, but also afforded interviewers the flexibility to ask follow-up questions to solicit further insight. The topic guide was developed by members of the research team, which included pharmacists, and researchers with expertise in conducting suicide and health services-based research.

### Data analysis

We conducted a thematic analysis in order to identify patterns of meaning across the data [16]. Due to the lack of prior research in this area, we used an inductive analysis approach in which findings were driven by the data [16, 17]. Five members of the multi-disciplinary research team (HCG, DL, CL, KLW, MS) contributed to the analysis. This provided a triangulation of views based on the different expertise of the research team, which included a registered practising pharmacist (PhD), a psychologist (PhD) with experience conducting suicide-focused and qualitative research, and pharmacy masters students. We followed the step-by-step guidance to data analysis as outlined by Braun and Clarke [16]. First, researchers read and re-read the corpus of transcripts to become familiar with the data. Second, five transcripts were coded individually (MS, KW, CL, HCG, DL) and then discussed in a group meeting. Through this discussion, a more extensive coding framework was developed that was used to code the entire data-set (HCG, DL), with additional codes generated as necessary. The coding process was iterative, and the first and second author (HCG, DL) met periodically to review consistency between coding. Any discrepancies between the application of codes were resolved by returning to the coded data to review and develop coding descriptions. We used NVivo version 12 Pro software to manage the coding process, which included definitions of all codes. Third, initial themes were developed through grouping codes based on their similarities and differences. Fourth, a series of group meetings were held to review and refine the themes to ensure that the thematic structure coherently reflected the dataset. No new codes were generated from the last three interviews, signalling that the data were saturated.

## Results

We conducted 25 semi-structured qualitative interviews with pharmacy staff. Eighteen participants were female and the full working age range (18–65+) was represented. Ten participants were dispensing/pharmacy assistants, eight were pharmacists, and the remaining seven were pharmacy technicians, pre-registration pharmacists or delivery drivers. Seventeen participants worked for a pharmacy that were part of a large multiple chains whilst eight worked independent pharmacies (Table 1).

**Table 1:** Summary of demographic characteristics of participants.

Demographic	Number (n = 25), Percentage (%)
**Gender**	
Male	7 (28%)
Female	18 (72%)
**Age (years)**	
<25	2 (8%)
25–34	8 (32%)
35–44	2 (8%)
45–54	8 (32%)
55+	5 (20%)
**Role in pharmacy**	
Pharmacist	8 (32%)
Pharmacy Technician	3 (12%)
Dispensing/healthcare assistant	10 (40%)
Pre-registration pharmacist	2 (8%)
Delivery driver	2 (8%)
**Type of pharmacy**	
Independent	8 (32%)
Multiple	17 (68%)
**Geographical location**	
Large town or city	11 (44%)
Small town	9 (36%)
Suburb	5 (20%)
**Situation**	
Shopping street	5 (20%)
Shopping centre/mall	2 (8%)
On its own	12 (48%)
Co-located with a doctor’s surgery	3 (12%)
Other	3 (12%)

**Fig 1:**
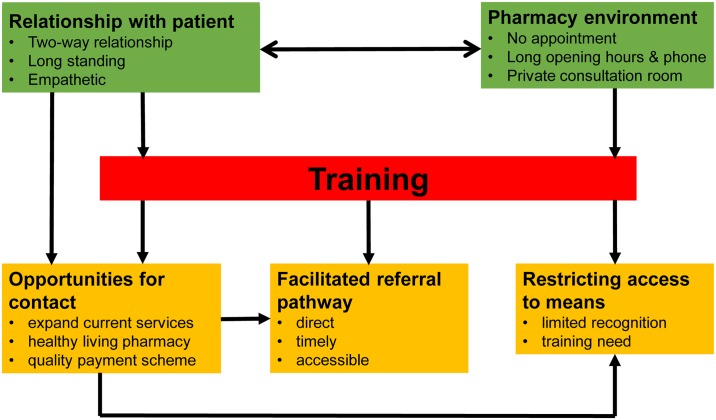
Conceptual model to depict the interrelationship between themes. A conceptul model to represent the role of community pharmacy teams in suicide and self-harm prevention as deduced from the emerging themes from this data. Pharmacy team persons are intergral to, and overarching in, the whole model.

Overall, community pharmacy staff were positive about their potential to contribute to suicide prevention. To realise this potential, they identified the need for appropriate training, commensurate with their role. Many, but not all, participants shared an example of how they had helped people who were contemplating suicide. We identified six key, interrelated themes to describe the current role of community pharmacy teams in suicide prevention and the potential for current activities to be enhanced to provide further opportunities for suicide prevention. A conceptual model to depict the interrelationship between themes is shown in Fig 1. The *Relationship with the Patient* and *Pharmacy Environment* were seen as platforms for engagement in suicide prevention pathways. With appropriate *Training*, it was felt that community pharmacy teams could contribute to suicide prevention by utilising existing *Opportunities for Contact* with patients, developing a *Facilitated Referral Pathway* and consider *Restricting Access to Means*.

### Relationship with patient

Participants reported good professional relationships and rapport with patients, especially with those who regularly visited the pharmacy. These relationships were often described as continuous and longstanding over many years. This was seen as beneficial as it both enabled patients to feel comfortable in approaching pharmacy teams; and allowed the pharmacy team to spot potential changes in patients with whom they had good rapport. This was evident in many of the examples where participants had helped a patient, who was already known to them, with suicidal thoughts or behaviours.

*“And I think there’s a massive role because our teams are all based in the community here*, *they know the people*, *we know our customers really*, *really well*. *Anybody who comes in*, *we’re on first name basis*. *So we have that relationship with them*. *We see them very*, *very often and we can recognise a decrease in symptoms*, *alarming symptoms*, *any sort of changes in presentation*, *we recognise*, *and we can help and support them quite quickly*.*”*

*(P19*, *Pharmacist)*

Participants described using this established relationship with patients to provide reassurance, conversation and a listening ear, whilst demonstrating empathy.

*“When they come in*, *there’s one lady in particular who can come in and she can be quite juddery*, *shaking and being very erratic and quite blunt*, *and you know straight away that she’s not having a good day*. *I always sort of go over and sit by her if the shop’s not too busy and just have a little chat to her*, *how’s she doing*, *anything I can help her with and stuff like that*.*”*

*(P16*, *Dispensing Assistant)*

It was acknowledged that pharmacy support staff may be more engrained into a particular pharmacy and community than the pharmacist, thus were perceived to be integral to developing and maintaining relationships.

*“In a community pharmacy you might have*, *today’s pharmacists might be qualified 30 years*, *tomorrow’s* six *months*, *you know*, *because they’re running on locums*, *or whatever*. *So*, *that’s where maybe training the regular staff might actually be more of a benefit than training a pharmacist”*

*(P8*, *Pharmacist)*

In contrast to the value of knowing the patient, the same participant suggested that some people might prefer speaking to staff whom they don’t know, to maintain anonymity.

*“And then if you’ve got the regular staff trained a lot of the times we’re…in my experience of working*, *regular staff tend to live in that area*, *which might be a good thing for some patients if they’re thinking about suicide or self-harm*, *but for others*, *who don’t want anybody to know*, *it’s a barrier”*

*(P8*, *Pharmacist)*

### Pharmacy environment

The layout and accessibility of the pharmacy were seen as facilitators for pharmacy team involvement in suicide prevention. Pharmacies were noted to have extended opening hours compared to general practices, could be reached via the telephone or in person, with no appointment required to speak to trained healthcare professionals and support staff.

*“Because we are accessible with patients and people*. *Most of them*, *the doctors are like open at 09*:*00*, *close at 18*:*00*. *Just walk in [here]*. *Staff are trained*, *I’m trained*. *Since we are accessible*, *I think we can attend [to] more people*.*”*

*(P11*, *Pharmacist)*

The community pharmacy was considered to be a safe place for confidential and sensitive conversations. The private consultation room was highlighted as a particularly suitable environment that staff had used, or could use, to talk to people about suicide.

*“[I] sat down in the consultation room talking to a patient who was very worried about everything that was going on in the world and that he wanted to harm himself*.*”*

*(P14*, *Pharmacist)*

Whilst the community pharmacy environment was generally viewed favourably, there were some barriers described such as funding for services and that pharmacies are busy places thus may have time and resource limitations.

*“It seems with community pharmacy if it’s not and this is really cynical but if it’s not something that gets paid for*, *you know*, *like a service then it usually doesn’t get done which and I’m not saying that that’s like*, *like a general thing*, *I just think that you’ve got a responsibility regardless of whether you’re going to get paid to do it*.*”*

*(P22*, *Pharmacist)*

Whilst the overall attitude of participants regarding having conversations about suicide was positive, a few concerns were raised. Some participants felt their colleagues might have reservations in relation to the scope of their job role, personal views, attitudes, lack of personal experience and potential stigma.

*“As a team we could do a lot more to help with mental health issues*, *spot things better*. *But there are people who would have to have training because they haven’t had experience of it*, *which is only right*. *If you haven’t got the experience you’re not going to know what to look for*.*”*

*(P16*, *Dispensing Assistant)*

### Training

Pharmacy staff relied upon personal experience to shape their approach to supporting patients about suicide and self-harm. This included their own experience or that of their family, or experience they had gained from voluntary work or pharmacy practice in other settings. In the absence of formal training and reliance on personal experience, some participants were unsure if they had acted appropriately.

*“I*: *So*, *do you have any training in suicide or self-harm awareness or prevention*?

*R*: *No*, *only what I’ve learnt through people that I know suffering with mental health and myself*.*”*

*(P15*, *Pharmacy Driver)*

*“I was happy to talk to him about it*, *I just didn’t know if I was doing the right thing or saying the right things*. *As you asked me before*, *I have not had any specific training*.*”*

*(P14*, *Pharmacist)*

No participants had undertaken any formal training on suicide prevention, including pharmacists in their undergraduate and preregistration training, but all welcomed this prospect.

*“I do believe that if we had more training in it then I feel like we could be a bit more effective*, *and I feel like with the mental health burden*, *I think pharmacists can produce some positive outcomes*.*”*

*(P2*, *Pharmacist)*

Whilst the *Relationship with patient* and *Pharmacy environment* themes identified a platform, training was recognised as a prerequisite to move towards the subsequent themes. Although participants recognised that there is stigma surrounding suicide, many lacked the knowledge of the recommended language to use when talking about suicide (e.g. they used committed suicide rather than died by suicide) [18]. Pharmacy teams had some knowledge about suicide but this was often incomplete or inaccurate. For example, one participant held the misconception that the suicide rate in the UK has increased, when overall rates have declined for the past two years [2].

*“I know that it’s [suicide] gone up*, *the statistics of it has definitely gone up*, *I would say in the last few years but I’m certainly not an expert on it”*

*(P23*, *Pharmacy Technician)*

### Opportunities for contact

As described in the *Relationship with Patient* theme, pharmacy teams described interacting with patients if they identified changes in behaviour, and providing an “open door” for patients to speak to the pharmacy team. There were existing opportunities for contact with patients which pharmacy staff reported using to interact with patients regarding suicide. They were predominantly based on knowing that the patient has a mental health problem, determined from the prescriptions for medication that the patients presented at the pharmacy.

*“It’s seen on the medical history that they were on anti-depressants and ask them if they see people regularly*, *how are the symptoms*, *how is their state of mind*, *at the moment*, *and do you think that you need help*?*”*

*(P13*, *Pharmacy Technician)*

Pharmacy staff identified a number of opportunities for increased interaction with patients about self-harm and suicide risk, if underpinned by training. These suggestions were based on expanding current initiatives. There were examples where the MUR service was used to facilitate conversations but participants acknowledged that these conversations could be maximised. The NMS was also seen as a service that could be expanded to facilitate conversations with populations at risk of suicide such as those with depression, as identified from antidepressant prescriptions.

*“I know you can’t say a hundred per cent about the patient but at least maybe you can start the conversation during your MUR or in some other way confident*, *by keeping the confidentiality of the patient you can help them and ask them to start chatting about their problem*.*”*

*(P18*, *Pre- registration pharmacist)*

*“NMS is another one because obviously if someone’s been newly prescribed an antidepressant…see*, *it’s not on one of…it’s not one of the drugs that you would target NMS for*, *but if someone’s been newly prescribed an antidepressant you should be having those types of conversation”*

*(P8*, *Pharmacist)*

Pharmacy staff suggested that suicide prevention efforts could mirror the approach taken by existing public health schemes, such as dementia friends and the Healthy Living Pharmacy concept. The inclusion of Healthy Living Pharmacy in the Quality Payments Scheme was also seen as providing an additional incentive.

*“Last year we’d done something for…it was LGBT [lesbian*, *gay*, *bisexual and transgender] I think it was and as soon as we put our certificate up that we’re gold service and we welcome everybody the amount of people that have noticed it*, *yes*, *yes*, *yes […] but if there was just like a poster up to say*, *they would notice it straightaway*.*”*

*(P20*, *Dispensing Assistant)*

*“like [you] have [a] dementia health learning champion*, *almost [introduce] like a mental health champion*, *trained to how they would start that initial discussion”*

*(P1*, *Pharmacist)*

*“I think they should be forced to do something so*, *you know*, *like with the healthy pharmacists or the quality payment schemes*, *they should be doing something about* …*mental health and that might mean*, *you know*, *running a campaign”*

*(P22*, *Pharmacist)*

### Facilitated referral pathway

Pharmacy teams were unsure who patients should be referred to and how to make this referral. In lieu of this knowledge, they heavily relied on signposting to the GP, by which they meant telling the patient to go to the GP. Sometimes pharmacy staff directly facilitated a referral by making phone calls on the patient’s behalf to the GP or utilised other formal care pathways that are in place for that specific patient (e.g. substance misuse key worker). Occasionally, this was extended to asking the patient if the pharmacy team member could contact a family member. Charitable organisations were also cited as potential support resources.

“It’s just basically if we find someone how would you deal with it but the only way we know how to deal with it would be to send them to the doctors.”

*(P25*, *Dispensing Assistant)*

*“We have referred people into MIND [mental health charity]*, *just for mental health support*. *We do also refer into Citizens Advice for benefit and financial if that’s what they need as well*, *if that’s a contributory factor*.*”*

*(P19*, *Pharmacist)*

The absence of a well-defined referral system was highlighted as a barrier to effective suicide prevention. It was deemed essential to establish clear, direct referral pathways to facilitate pharmacy teams to support people. This was identified as a knowledge gap in *Training* and is critical to support utilisation of *Opportunities for Contact*. Training must also be evidence-informed and include facts about suicide and communication skills. A few participants suggested the creation of a Standard Operating Procedure (SOP) would help to streamline referral processes.

*“there’s not very good referral systems*, *so it would be good if a patient came in and they have any mental health problems or you are feeling suicidal*, *or have been self-harming*, *I could call someone*, *you know*, *I know I could call someone*, *almost like log it*, *that this had been an issue*. *And then it would*, *they would get seen*, *do you know what I mean*?*”*

*(P1*, *Pharmacist)*

In addition to one-way referral from pharmacy to other providers, a gap in the two-way communication between community pharmacy teams and other healthcare professionals was evident. Improvement is needed in two-way communication particularly regarding patients who may have been prescribed small quantities of medication due to increased vulnerability for self-poisoning.

*“I also know that I have seen [once]-weekly scripts and daily scripts for people who I’m guess*[ing] *are at risk*, *I don’t know whether it’s something that they have maybe tried to commit suicide or maybe they are just at risk and they have ended up on daily scripts or weekly scripts*, *cause we have dispensed those*. *These are all things that I sort of put together and sort of come to this conclusion*.*”*

*(P14*, *Pharmacist)*

### Restricting access to means

Participants rarely spoke of the methods of suicide or self-harm. The few mentions of methods erred towards violent methods more so than poisoning.

*“We have another patient that we’ve had phone calls where his is threatening to throw himself out of the window*, *he has threatened to set his flat on fire*, *he goes into the local mental health unit but then discharges himself within a day*.*”*

*(P25*, *Dispensing Assistant)*

There were some acknowledgements that medicines supplied against prescription or sold to patients may be used for intentional self-poisoning. However, there also seemed to be a perception that patients would be unlikely to use medication supplied by the pharmacy to harm themselves.

*“I*: *do you think about medicines that people are dispensed*, *do you think they’re ever used in poisoning*?

*R*: *No*, *I wouldn’t say so*, *not for the medication that they’re on*. *[…] I mean*, *this person was on a dosette tray so*, *obviously*, *he could only punch one out*, *or if he got to the point that he couldn’t do it anymore*, *he would take a lot*. *But*, *he would ring me and tell me*, *and I’d say*, *well*, *go to A&E*. *You know*, *a couple of times when he’s rang*, *he’s said*, *but*, *I’m sat here*, *can you come*, *and I can’t do that*.*”*

*(P24*, *Dispensing Assistant)*

Conversely, some pharmacy staff referred to examples where limited quantities of medication were prescribed to individuals thought or known to be at high risk of poisoning. This is also discussed above in the relation to ineffective two-way communication with other healthcare professionals.

*“it was only until the staff told me that this patient is like this that’s why we do this*, *we give them a weekly tray because they self-harm they can over-medicate themselves”*

*(P21*, *Pre-registration Pharmacist)*

Pharmacists who described concerns regarding the use of prescribed medication in poisoning often had multisector experience, such as the ambulance service or mental health pharmacy.

*“I think as pharmacists regardless of which area or sector you work in*, *you know*, *one of the main* …*not main but one of the ways people*, *you know*, *attempt suicide is that they stockpile medication*, *you know*, *so*, *you know*, *that’s a role for a pharmacist [*…*] but you get very little*…*you get some pharmacists*, *oh*, *don’t want to deal with that*, *you know*, *so* …*I think it’s to do with the undergraduate course*, *you just don’t get* …*you don’t get access to*, *you know*, *mental health*. *Maybe it’s changed*, *you know*.*”*

*(Pharmacist*, *P22)*

## Discussion

We present the first in-depth qualitative interview study examining the current and potential role of community pharmacy teams in self-harm and suicide awareness and prevention. We developed a conceptual model to illustrate the current role of community pharmacy teams (*Relationship with Patient*, *Pharmacy Environment)* and how, with the support of *Training*, this could be enhanced to maximise the interactions that pharmacy teams are already having with people about suicide *(Opportunities for Contact*, *Facilitated Referral Pathway*, *Restricting Access to Means)*. Our recommendations for practice are summarised in Table 2 and used as subheadings to direct the reader through this discussion.

**Table 2:** Summary of recommendations for pharmacy practice and research.

Recommendation	Details
Evidence-informed training programe available for all community pharamcy staff.	The whole pharmacy team should be trained in: suicide statistics, dispelling myths, communicating about suicide and navigating effective referral systems. This training programme should be evidence informed and robust measures of intial and continued efficacy should be integrated into systems.
Maximise suitablity of pharmacy environment.	The pharmacy environment, including availability of private consulation rooms, already supports conversations about suicide and self-harm. Patients should be made aware that the pharmacy is a place where one can talk about suicide.
Design of an effective and accessible referral system.	Referral systems should be co-designed with relevant stakeholders. Ideally, they should enable direct referral by pharmacy teams to an appropriate provider at all times during the pharmacy’s opening hours.
Optimise the current pharmacy contract to provide incentivised opportunities for suicide prevention.	Current features of the community pharmacy contract and Quality Payments Scheme could be a vehicle for suicide prevention activities. For example, public health campaigns, mental health champions and formal adaptations to Medicine Use Review (MUR) and New Medicines Service (NMS) schemes. It is essential that the current and potential role of pharmacy teams in suicide prevention is researched from the perspective of the patient.
Explore the involvement of medication in suicide and self-harm.	Pharmacy teams seldom mentioned the provision of medication used in poisoning. Further qualitative and epidemiological research could unpack this.

### Maximise suitability of pharmacy environment

The pharmacy setting was considered to provide an opportunity to talk about suicide. This encompasses the strong relationships that pharmacy teams have with patients and the physical environment of the pharmacy. Correspondingly, these components comprised the “accessibility for confiding theme” in Murphy et al.’s thematic analysis of open-ended survey questions about community pharmacists’ experience of caring for people at risk of suicide in Canada and Australia [14]. Participants in our study often used their instinct, personal or professional experiences to inform conversations. A good, long-term relationship between pharmacist and patient was cited as a mechanism to achieve resolution of problems related to mental health and medication in a mental health and addictions programme in Nova Scotia (Bloom Program). The private consultation room was the main location for these discussions [19]. In the UK, despite the requirement for a private consultation rooms in the community pharmacy contract, patients are often unaware that this facility is available [20]. Conversely, pharmacy staff described using these spaces to have sensitive conversations with patients. A sign to notify patients that the private consultation room is available for patients to discuss mental health-related issues with pharmacy staff could be useful.

### Evidence-informed training

In order to maximise the involvement of community pharmacy teams in suicide prevention, *Training* for the whole pharmacy team was considered essential. Neither registered nor unregistered staff reported participation in training; this included as part of pre-registration pharmacy degree programmes. Similarly, in a survey of 501 community pharmacy staff in North Carolina, just 7% reported having training in suicide prevention yet 89.6% said that they would be willing to participate in training. (15) The GPhC sets the *Future Pharmacists*: *Standards for the initial education and training of pharmacists* in Great Britain [21]. This document does not explicitly refer to suicide prevention training. Although we know of local examples, there is no published review of the training available to pharmacy staff or trainees in the UK. In the USA, a recent scoping review identified 16 suicide prevention training programmes that included pharmacists or pharmacy students in their target audience [22]. Most of these resources contained information on suicide statistics, suggested referral routes and communicating about suicide. This broadly maps to the training needs identified in our study-both explicitly and as interpreted from knowledge gaps. The review identified Washington State as the only state which mandates suicide prevention training for pharmacists [22]. By the end of 2018, all pharmacists in Washington were required to have completed a 3 hour-long training package which covers screening and referral, under the Matt Adler Suicide Assessment, Treatment and Management Act of 2012 [23]. In their scoping literature review of research into pharmacists and suicide prevention, Murphy et al. identified four research studies which explored the impact of training on pharmacy teams. [13] Evaluation of these training programmes measured changes in attitudes and knowledge [13, 22]. Whilst it is extremely difficult to measure the direct impact of training on rates of suicide, related outcomes should be measured, such as monitoring referrals from community pharmacy following conversations with patients about suicide.

### Effective and accessible referral system

Training in suicide and self-harm prevention is one the listed competencies in the Self-harm and Suicide Prevention Competence Frameworks [8]. In the same Frameworks, “liaison with others” is a listed competency. The converse, gaps in communication, was cited by our participants as a barrier to pharmacy teams understanding a person’s suicide risk. This was based on examples where they were not informed about measures aimed at reducing suicide risk that were employed by the GP or other professionals involved in their care, for example restriction of the quantity prescribed. Furthermore, the need for *Facilitated Referral Pathways* was viewed by pharmacy staff as essential. For these to be effective, they need to be localised, accessible and ideally permit direct referral from pharmacy. Although some other referral options were mentioned, pharmacy teams heavily rely on referral to GPs. However, GPs report frustrations at gaps in communication reported between GPs and pharmacy staff in this study, GPs also reported communication gaps with specialist services [24]. One of the benefits of community pharmacies identified by our participants was the out-of-hours accessibility. In order to design appropriate referral systems for community pharmacy teams to use, input is required from all local relevant stakeholders and should enable the pharmacy team to be able to provide referral at they are open, for example late at night, at weekends and on public holidays.

### Optimisation of the current pharmacy contract

Community pharmacy staff identified various *Opportunities for Contact*, which build on existing services and processes and could be achieved if training and referral pathways are in place. There were examples of pharmacists using the MUR service as a platform to talk about suicide. There have been recommendations for follow-up and monitoring in MURs [25]. The Bloom Program, previously described [19], could fit this specification from a mental health perspective. However, this would require remuneration, something seen as a barrier by participants. Similarly, some participants suggested the inclusion of antidepressants in the NMS. The same recommendation was made in a qualitative synthesis of initial antidepressant use in the UK and Australia [26]. Further research is required to evaluate the safety and cost-effectiveness of this potential expansion. In any such design, it would be wise for suicide training and referral processes to be activated, because people with depression have a greater risk of suicide compared to those without [1].

Some participants identified the public health campaigns associated with Healthy Living Pharmacies to be an opportunity to publicise that pharmacy staff can talk about suicide. This corroborates with Firth et al.’s qualitative study which noted that engagement between pharmacy staff and users improved when the pharmacy was a Health Living Pharmacy [27], which, as previously mentioned, is a criterion of the Quality Payment Scheme [11]. Some of our participants saw this scheme as a way to leverage funding in suicide prevention, particularly in relation to having a dedicated “champion” in each pharmacy who actively promotes and coordinates a particular public health message. They suggested these creative ways to include suicide prevention in their day-to-day activities. Participants spoke of instances where patients had disclosed suicidal thoughts. Given that GPs have reported having no contact with some patients prior to suicide [24], pharmacy teams could provide an alternative point of contact if appropriately upskilled and embedded within referral pathways. Participants highlighted the need to include the whole pharmacy team in training, as anyone could be involved in conversations about suicide. Training has been suggested to improve the confidence of pharmacy staff in talking about suicide [28]. There was still some uncertainty about whether everyone would feel comfortable doing so, and some concerns regarding stigma. A recent survey of pharmacists practicing in Canada and Australia showed low but differing levels of acceptance of stigmatising terms when describing a person who has died by suicide [29]. The role of pharmacy teams from the perspective patients is unknown, (13) and is a significant research gap.

### Explore the involvement of medication in suicide and self-harm

Participants in this study tended to see their involvement with patients about suicide to be related to the social support aspects. They sometimes used medicines, such as anti-depressants, to identify people who might be at risk of suicide based on the condition they were been treated for. There were no mentions of exploring suicide as an adverse event of medication and few mentions of medicines used in overdose.

Pharmacy staff rarely acknowledged that they may have a role in restriction of access to means of suicide through provision or restriction of medication.

Conversely, gatekeeping of medication was identified as a subtheme from Murphy et al.’s qualitative analysis of questionnaire responses from pharmacists practising in Australia and Canada [14]. The differences between ours and Murphy’s results could be due to differences in medication availability over-the-counter in the three countries. However, they cited examples where the pharmacist explored patient’s intentions to use medications in poisoning including where pharmacists refused to dispense medication due to these concerns [14]. An alternative possible explanation for this difference is that pharmacy staff lack awareness regarding the prevalence of self-poisoning as a form of self-harm and suicide. Poisoning is the second most common method of suicide [2], most commonly reported method of self-harm to General Practices in the UK [30], and accounted for 1% (n = 215,735) of emergency department attendances in England in 2017/18 [31]. Some of the medications involved in both accidental and intentional poisoning deaths [32] can only be obtained legally from a pharmacy with a valid prescription, although some may be sourced illicitly or prescribed to someone else. Further research is need to explore the relationship between medication access and suicide, both from the patient perspective and an epidemiological standpoint. This may provide an opportunity to inform policy in suicide prevention.

### Strengths & limitations

This is the first in depth qualitative interview study to explore the current role of community pharmacy teams in suicide prevention, and what staff perceive as the potential enhancement of this role. Furthermore, our multidisciplinary team has enabled us to view the data from various perspectives and come to a consensus on the defined themes. Findings from this study are derived from staff who volunteered to be interviewed about suicide prevention therefore there will be a degree of selection bias. It is possible that these individuals may have a particular interest in suicide prevention and thus that these findings may not reflect the views of staff who are less supportive of contributing to suicide prevention. Indeed, some of the participants elected to discuss their personal experience, although they were not explicitly asked about this.

### Future research

Our research has elicited clear lines of enquiry ripe for future research. To complement our findings, it is essential to understand the opinion of people with lived experience and other stakeholders of the role that community pharmacy teams could or do play. Little was discussed about safeguarding, despite this being an obligation of community pharmacy teams which could have substantial overlap with suicide prevention. Further epidemiological and qualitative studies are required to understand the relationship between medication access and suicide.

### Conclusion

Community pharmacy teams provided examples of when they have given support to someone thinking about suicide. The strong relationships that many patients have with pharmacy teams coupled with the accessible and safe pharmacy environment provided a platform for these disclosures. Generally, community pharmacy teams are willing to have an enhanced role in suicide awareness and prevention if they receive adequate training and are supported by participation in a facilitated referral pathway.

## Supporting information

S1 File Topic guide and interview schedule. (PDF)

S2 File CORE-Q checklist compliance. (PDF)
