# Supplementary material for: Current and potential contributions of community pharmacy teams to self-harm and suicide prevention: A qualitative interview study
Source: PLoS One. 2019 Sep 9;14(9):e0222132. doi: 10.1371/journal.pone.0222132 (PMC6733435; doi:10.1371/journal.pone.0222132)
Supplement: S1 File — (PDF) [file pone.0222132.s001.pdf]

Awareness and prevention of suicide and self-harm in community pharmacy: an exploration of current and potential roles

Topic Guide and Interview Schedule

Introductions

Introduce self & reassure that participant does not need to answer questions if they do not wish to, and the interview can be stopped at any time. Check if the participant has any questions before you comment.

General comment regarding confidentiality:

I want to assure you that whatever we talk about will be confidential between you, I and the research team; and all data will be anonymised. However, if I feel that there is a risk to you or others, or any unsafe practice or fitness to practise is disclosed, I will have to report this accordingly. If I feel the need to do this, I will discuss this will you.

Establish participants' current role in community pharmacy.

1. Can you briefly describe the community pharmacy that you work (mainly) in?
   -geographical location & situation
   -how busy?
2. Please describe your role in community pharmacy.
3. What do your usual day-to-day activities entail?

Establish participant's knowledge on suicide and self-harm awareness and prevention generally.

4. Can you tell me a little bit about what you know about suicide and self-harm?
   Prompts: -who it affects
   -how common it is
   -who cares for these people
5. Do you have any training in suicide or self-harm awareness or prevention?
6. In your local area, do you know how people who have self-harmed or have plans or thoughts of suicide are looked after?

Participant examples of when they have dealt with someone who had thoughts or plans of suicide or self-harm, or had self-harmed, and presented to the pharmacy (if any).

7. Do you have any experience of looking after someone who has thoughts or plans of suicide or self-harm, or has self-harmed, in your pharmacy?
   Prompts:
   -describe what you did?
   -how did you feel?

Explore the potential role of community pharmacy teams in suicide and self-harm awareness and prevention.

8. In your opinion, could community pharmacy teams do anything differently to help people who have thoughts or plans of suicide or self-harm, or have self-harmed?

Explore perceived training needs for community pharmacy teams on suicide and self-harm, if any.

9. Should community pharmacy teams have any additional training on suicide and self-harm?
10. (if answers yes to 9) What sort of training should community pharmacy teams have?
    Prompts: -content-extent and type
    -delivery e.g. face-to-face, e-learning

Invite participant to comment any other related issues that they wish to discuss.

Participants will then be asked if they have any other comments that they wish to add relating to the topic of suicide and self-harm awareness and prevention in community pharmacy. The interview will then be closed and the participant will be provided with a debrief sheet and asked to sign to confirm receipt and understanding of this sheet.
